# Breath Analysis in Disease Diagnosis: Methodological Considerations and Applications

**DOI:** 10.3390/metabo4020465

**Published:** 2014-06-20

**Authors:** Célia Lourenço, Claire Turner

**Affiliations:** Department of Life, Health & Chemical Sciences, Chemistry and Analytical Sciences, The Open University, Walton Hall, Milton Keynes, MK7 6AA, UK; E-Mail: celia.lourenco@open.ac.uk

**Keywords:** volatile organic compounds (VOCs), trace gas analysis, volatile biomarkers, metabolomics

## Abstract

Breath analysis is a promising field with great potential for non-invasive diagnosis of a number of disease states. Analysis of the concentrations of volatile organic compounds (VOCs) in breath with an acceptable accuracy are assessed by means of using analytical techniques with high sensitivity, accuracy, precision, low response time, and low detection limit, which are desirable characteristics for the detection of VOCs in human breath. “Breath fingerprinting”, indicative of a specific clinical status, relies on the use of multivariate statistics methods with powerful in-built algorithms. The need for standardisation of sample collection and analysis is the main issue concerning breath analysis, blocking the introduction of breath tests into clinical practice. This review describes recent scientific developments in basic research and clinical applications, namely issues concerning sampling and biochemistry, highlighting the diagnostic potential of breath analysis for disease diagnosis. Several considerations that need to be taken into account in breath analysis are documented here, including the growing need for metabolomics to deal with breath profiles.

## 1. Introduction

The developments in diagnostic methods and monitoring technologies have focused on blood and urine analysis for clinical diagnostics. The contemporaneous technological advance in analytical techniques allows the measurement of volatile organic compounds (VOCs) emitted from clinical samples, such as exhaled breath, urine, blood, serum, sputum, and faeces. In spite of its advantages, diagnostics based on VOCs profiling is not yet widely used in clinical practice [1].

During the last decades, Selected Ion Flow Tube Mass Spectrometry (SIFT-MS) [2], Proton Transfer Reaction Mass Spectrometry (PTR-MS) [3], and Gas Chromatography–Mass Spectrometry (GC-MS) [4] with thermal desorption or solid-phase micro extraction (SPME) have been widely used for medical research. SIFT-MS and PTR-MS analytical techniques have been developed for potential medical applications, by using breath analysis, urine analysis, faecal analysis, *in vivo* human skin studies, and *in vitro* cell cultures [5]. SIFT-MS and PTR-MS were developed for real-time, on-line detection and quantification of trace gases in air, with a high sensitivity and wide dynamic range. 

Breath is an obvious matrix for analysis of VOCs, as the VOCs are generated within the body, travel around the body via the blood and then they can cross the alveolar interface and appear in exhaled breath, being measured at trace concentrations in the parts-per-million by volume (ppmv) and parts-per-billion by volume (ppbv) levels or lower [6]. Although the trace compounds produced in the oral cavity do not necessarily enter the blood stream, they do appear on exhaled breath. Analysis of the concentrations of VOCs in breath with an acceptable accuracy can provide an indicator of metabolic status, allowing a distinction between healthy and diseased states. Thus, these techniques have the potential to detect diseases in their early stages, non‑invasively and painlessly.

Michael Phillips has been a pioneering breath researcher for more than thirty years, providing evidence of the presence of identifiable VOCs in the breath related to lung and breast cancer [4,7,8]. Later on, Anton Amann organized the International Association of Breath Research (IABR), followed by the annual international meetings on Breath Analysis, starting in 2004. 

The scientific community is motivated to study all parameters which influence the appearance of VOCs in human breath. For that reason, there are crucial points that should not be neglected, such as standardised methodology for breath sampling and analysis. Factors, such as pulmonary gas exchange and contamination, should be taken into account during the development of breath sampling procedures.

Breath analysis requires elaborate methods of data analysis including multivariate statistical methods, which are applied to show statistically significant differences between the groups (healthy/disease). In addition, there is often little agreement between studies as to which VOCs constitute an appropriate discriminating set. Despite these facts, mass spectrometric analytical techniques have proven to be suited for the challenge and are well suited to both biomarker discovery and whole spectral profiling.

## 2. Techniques for Breath Analysis

Biomarkers research relies on analytical methods (Table 1) that offer high sensitivity, precision and resolution. The on-line, real-time analytical techniques SIFT-MS and PTR-MS exhibit limit of detection ranging from ppbv to pptv, making them ideally suited to breath analysis [9]. Proton transfer reactions occur in both techniques in a chemical ionization process that allows a very efficient ionization for many organic compounds in the gas phase. Product ion generation in SIFT-MS and PTR-MS is managed using chemical ionization, arising from ion-molecule reactions rather than electron impact or photoionization, with much less fragmentation of the molecules. Thus, these techniques are called *soft* ionisation techniques. SIFT-MS and PTR-MS are well suited to direct, real-time MS profiling without pre-concentration and with limit of detection ranging from ppbv to pptv. Hence, MS data is well placed for this type of analysis, by means of using sophisticated detectors enabling unequivocal real time quantification of volatile organic compounds, with high sensitivity, precision and resolution. The MS data sets are quite simple, easy to handle, including numerous variables perfectly suited for multivariate statistics. In contrast, a major advantage of chromatographic methods is its very high sensitivity due to sample concentration. In addition, the existence of extensive compounds libraries makes compound identification much easier than in SIFT-MS and PTR-MS. 

**Table 1:** A comparison of the characteristics of the available breath research techniques.

Analytical Method	Mode of operation	Limit of detection (LOD)	Sensitivity	Specificity
SIFT-MS	Direct/Real time	ppbv	High	High
PTR-MS	Direct/Real time	pptv	High	Medium-High
IMS	Real-time	ppbv	Medium	Medium
Sensor arrays	Reference to a database	ppbv	Medium	Medium
GC-MS	Pre-concentration	pptv-ppbv	Very-high	Very-high
LAS	Real-time	ppbv	High	High

Other techniques are also widely used, including laser absorption spectroscopy (LAS), ion mobility spectrometry (IMS), and electronic noses containing a variety of gas sensors and semiconductor-based sensor arrays, although gas sensors are often much less sensitive, usually lack specificity, and are prone to drift. There are also difficulties in inter-device reproducibility. Concerning the IMS technique, the ions are generated by a radioactive strip and they are separated according to their mobilities through the gas, which is usually air at atmospheric pressure [10]. Such devices are not operated at high vacuum conditions and therefore ion-molecule collisions occur, limiting the speed of the ions along the drift tube. Hence, the number of ions reaching the detector is lower compared to the theoretical value. This is an important factor limiting the sensitivity of IMS. During the last ten years it has been applied in medical research, such as detection of skin volatiles [11], detection of volatiles in exhaled breath of patients with lung cancer [12], and determination of anaesthetics concentration in exhaled breath [13]. For the LAS technique, the amount of light absorbed by a sample is related to the concentration of the target specie in the sample. The LAS-based technique cavity ringdown spectroscopy (CRDS) has been successfully applied to measure NO concentration in exhaled breath [14]. This technique enables quantification of volatiles in exhaled breath down to below parts-per-billion by volume levels. It is particularly useful for monitoring purposes, and, recently, the exhaled breath of healthy volunteers was assessed by CRDS [15].

### 2.1. SIFT-MS

The SIFT technique was conceived and developed by N. G. Adams and D. Smith, in 1976 [16], for the study of ion-neutral reactions at thermal interaction energies [17]. Initially, it was developed to satisfy the need of kinetic data on gas-phase ion-neutral reactions observed in cold interstellar clouds [18]. In 1996, it became a method focused on real-time, on-line analysis of volatile trace gases of biological origin with medical applications [2,19,20,21], such as clinical diagnosis, therapeutic monitoring, and physiological studies [6,21,22,23]. With the SIFT–MS technique, it is possible to identify and differentiate isomers by using three different precursor ions (H_3_O^+^, NO^+^, O_2_^+^) and applying full-scan mode [24]. Compared to PTR-MS, SIFT-MS is less sensitive due to the existence of a mass filter, which selects the precursor ion to be used according to their mass-to-charge ratio. A clear advantage of SIFT-MS is that no electric field is employed and it is therefore possible to carry out ion-molecule reactions under thermal conditions where the kinetic behaviour is well known. Instead, the precursor ions are produced by electrical discharge, selected by a mass filter according to mass-to-charge ratio, and injected into a fast-flowing carrier gas (Helium), being thermalized [25]. Since the quantification is based on well-understood underlying physics and ion chemistry (in-built kinetics library), there is no need for regular calibration, in contrast to PTR-MS [26].

### 2.2. PTR-MS

Proton transfer reaction mass spectrometry (PTR-MS) was developed in the mid 1990s by Lindinger and co-workers [27]. This technique allows real-time, on-line determination of absolute concentrations of volatile organic compounds, with detection sensitivity greater than SIFT-MS. The first PTR-MS instruments developed only used H_3_O^+^ as precursor ion, a disadvantage compared to SIFT-MS. Currently, the latest instruments use a switchable reagent ion capability, alternating between the three precursor ions, H_3_O^+^, NO^+^, and O_2_^+^ like SIFT-MS [28]. There are usually overlapping ions in clinical sample headspace and the use of PTR–MS equipped with a time-of-flight (TOF) mass analyzer improves mass resolution to assist ion identification [29,30].

PTR-MS employs an electric field, *E*, along the flow tube axis to increase the velocities of the ions. The change of the ratio E/N, where *N* is the number density of the drift tube buffer gas molecules, will affect the reagent ion hydration and product ion fragmentation. Under normal operating conditions E/N is in the range 120–130 Td, representing a compromise between reagent ion hydration on the one hand, and molecular (product) ion fragmentation on the other. The electric field also prevents the formation of substantial quantities of cluster ions. In contrast to SIFT-MS, PTR-MS operates at higher effective temperatures and the underlying ion chemistry is often not known [31,32]. 

The instrument is much shorter, due to the existence of a shorter drift tube, has a typical length 10–20 cm, and, consequently, the pumping system is reduced in size, making PTR-MS suitable for transport. However, the latest advances in SIFT-MS have surpassed this issue, in which the instrument has become much smaller and suitable for transport [26]. 

The recent advances in PTR-MS technology have demonstrated a diverse range of applications, especially for breath gas analysis [33]. 

### 2.3. Electronic Noses and Semiconductor-Based Sensor Arrays

In order to measure different VOCs, many applications have combined various sensors and materials into a single array, leading to the development of an “electronic nose” [34]. Sensor technology has been used for many years in clinical testing. Currently, the goal consists of finding materials with high sensitivity and good selectivity to the VOCs to be detected. Up to now, the existing materials are mostly conductive polymers, semiconducting metal oxides, or a combination of the two [35]. Unique sensors, based on nanoparticles appear as a reliable alternative tool for breath analysis, proving to be inexpensive and easy-to-use. The quartz crystal microbalance (QCM) and the surface acoustic wave (SAW) device are mass-sensitive sensors which have been used in breath analysis [36]. In QCM, gas molecules are adsorbed on the crystal’s surface during sensor exposure to a gaseous medium, changing its mass and resonant frequency. A selective adsorption of the gas mixture strongly influences the degree of crystalline order and by the nanostructure boundaries. The polymer overall characteristics, length, planarity of the conjugate polymer chain, and the side chain composition may influence the polymer conductivity [35].

Recently, Cr- or Si-doped WO_3_ nanoparticles have shown high sensitivity to acetone, leading to the development of a portable chemo-resistance sensor suitable for real-time breath acetone detection [37]. Breath acetone concentration of five test persons at rest or during physical activity was measured and compared to that measured by PTR-MS. Si-WO_3_ sensors were selective to acetone in realistic conditions (90% Relative Humidity), and able to detect differences in breath acetone concentrations between 880 to 980 ppb, in agreement with PTR-MS measurements. 

However, the combination of specificity, selectivity, robustness in operation, reproducible manufacturing uniformity, and long-life stability is not offered by current sensors at an acceptable cost level. These technologies are unable to identify individual compounds, although they can be used to compare samples to see whether they have similar VOC profiles.

## 3. The Challenge behind the Method

Volatile compounds in breath are produced by metabolic processes at various organs and places in the body, in the oral cavity by bacterial infections, by bacteria in the gut or both. However, is likely that many are not biochemically produced in the body and predominantly come from environmental exposure.

Breath analysis started in the 1970s when Linus Pauling and co-workers detected over 200 different VOCs in human exhaled air and in urine headspace by gas chromatography [38]. Apart from the major components of breath, such as acetone, isoprene, *etc*., many trace compounds present are not of endogenous origin [39,40]. A large variety of trace gases exist in ambient air and these can be taken up via inhalation and skin absorption; the source of others is through ingestion. Hence, some of the trace compounds in exhaled breath, perhaps the majority of them, will be exogenous, and these need to be distinguished from the truly endogenous compounds [40,41]. The endogenous compounds found in human breath, such as inorganic gases (e.g., NO and CO), and VOCs (e.g., isoprene, ethane, pentane, acetone) can be measured directly; other typically non-volatile substances, such as isoprostanes, peroxynitrite, or cytokines, can be measured in breath condensate [39]. These non-volatile substances are supposed to be present in exhaled breath as aerosol particles. 

Background air VOC concentrations are an issue. The concept of alveolar gradient proposed by Michael Phillips [42], defined as *the abundance in breath minus the abundance in room air*, for substances having higher inspired than expired concentrations has been proposed to deal with this, however it, does not properly account for the background air a subject breathes. This has been demonstrated by the work of many researchers, including Schubert [43] and Španěl [44]. Indeed, it has been shown [43] that this does not lead to quantitative results; instead they suggested that when inhaled (ambient) concentrations of compounds are greater than 5% of the exhaled concentrations, exhaled concentrations cannot be correlated with blood levels with confidence. The levels detected in breath will depend on many factors, including the concentration in ambient air, the duration of exposure, the solubility and partition co-efficient into tissues, the mass and fat content of the individual, as well as the underlying endogenous concentration. Sample procedures for breath analysis have many advantages over traditional blood analysis of compounds suspended in or dissolved in blood: they are painless and non-invasive, easy to perform, inexpensive, and the results are available immediately for therapeutic assessments [36]. The exhaled air matrix is less complex than that of blood or other body fluids [41]. However, storage of blood is generally easier than breath. Traditional blood analysis typically involves measuring the concentrations of specific salts, proteins or other non-volatile components. However, sampling procedures for blood analysis are stressful for patients and a non-invasive sample, such as breath, is, therefore, preferable.

### 3.1. Mouth- vs. Nose-Exhaled Breath

The challenge of breath analysis for clinical diagnosis and therapeutic monitoring lies in the identification of endogenous volatile compounds present in mouth-exhaled breath which are potential markers of diseases, and which reflect levels in the systemic circulation. Many of these trace volatile compounds may be produced in the airways, the oral cavity by bacterial infections, by bacteria in the gut, and also emitted from mucus, saliva and aerosols created in the respiratory tract. Phillips and co-workers performed a pilot study by GC–MS for detecting VOCs in breath associated with oral malodour [45]. However, this technique has some limitations since the lower molecular weight VOCs may not be detected due to the sorbent trap selectivity for two carbon atoms or more. 

A sampling device of breath exhaled via the mouth, nose, and air in the mouth cavity was developed by Smith and co-workers [46]. Studies were performed using SIFT–MS to evaluate the concentration of mouth- and nose-exhaled breath, in order to understand the biological origin of several VOCs [46,47,48]. Ammonia in the exhaled breath is largely generated in the mouth [47] as is ethanol [46] and hydrogen cyanide [46]. This has been demonstrated through showing that the levels measured in the nose exhalations are much lower than those observed in the mouth exhalations. Very low concentrations of propanol and acetaldehyde in exhaled breath appear to be partially systemic and partially mouth generated [46]. Acetone, methanol, and isoprene showed similar profiles for mouth- or nose-exhaled breath [46], indicating that these compounds are totally systemic. However, methanol may be ingested by food intake or drink; hence methanol concentration in breath may not be totally produced through the human biochemistry. Thus to avoid the possibility of contamination of the endogenous VOCs through mouth flora, taking breath from the nose is desirable. 

A recent study using solid-phase micro-extraction of bacterial cultures demonstrated that several compounds detected in mouth-exhaled breath are produced by anaerobic bacteria in tongue biofilms [49]. In addition, poor oral hygiene can be a confounding factor leading to production of ammonia from urea or ethanol from sugars, thus, increasing VOCs concentration in mouth-exhaled breath [50].

Furthermore, volatile compounds may be produced by bacteria in the gut, transported to and excreted by the lungs [51]. *Helicobacter pylori* living in the human stomach release VOCs that can be detected in mouth-exhaled air [52].

The analysis of mouth- and nose-exhaled breath following ingestion of different doses of alcohol [53] at different concentrations in water was carried out using SIFT-MS by Smith and co-workers. They determined how the volume of ingested liquid influenced the gastric retention and degradation of ethanol. This showed the fraction of ethanol ingested and, consequently, the fraction which enters the blood stream, which in turn is diluted in exhaled breath. The decay of breath ethanol has been followed and measured in mouth- and nose-exhaled breath. The authors suggested that saturation of the liver enzymes have an important role in managing the decay of breath ethanol. Additionally, has been shown that gastric retention clearly results in a slower release of ethanol into the gut.

### 3.2. Physiological Levels of Volatiles

It is well known that human breath is a complex matrix of volatile organic compounds, non-volatile organic compounds (aerosol particles), and inorganic compounds. In order to develop a diagnostic breath test, ready to be used in clinical practice, it is necessary to unravel the baseline physiological levels of volatiles present in human breath, and their relationship with age, gender, ethnicity, and metabolic changes in the body.

#### 3.2.1. Common Breath Metabolites

Exhaled breath consists largely of unmodified (inhaled) nitrogen (somewhat less than 74% by volume if water vapour is included; 79% of the permanent gases) and argon (about 1%), oxygen (reduced from 21% inhaled to about 15% exhaled), carbon dioxide (about 5%), and water vapour (saturated at 37 °C -about 6% [54]).

Along with these major gases and vapours, there are many endogenously formed gaseous volatile metabolites present at trace levels variously measured in parts per million (ppmv), parts per billion (ppbv), and even parts per trillion (pptv).

Diskin and co-workers developed an initial study by SIFT–MS, measuring concentrations of the common breath metabolites ammonia, acetone, isoprene, ethanol, and acetaldehyde in the breath of five healthy subjects over a period of 30 days [40,55]. The mean concentrations were calculated, and meaningful distributions obtained for ammonia, acetone, isoprene, and ethanol. 

Later on, Turner and co-workers [56,57,58,59] performed longitudinal studies of the common metabolites ammonia, acetone, methanol, ethanol, propanol, acetaldehyde, and isoprene in the breath of 30 healthy volunteers over a six-month period, using SIFT–MS. Thus, the biological variability was assessed and the concentration distributions for these metabolites have been determined on-line in single breath exhalations and showed to be a log normal distribution for these metabolites. Ammonia was shown to be a major breath metabolite with a geometric mean of 833 ppb, followed by acetone (477 ppb), methanol (461 ppb), ethanol (112 ppb), isoprene (106 ppb), propanol (18 ppb), and acetaldehyde (22 ppb) [56,57,58,59]. Nevertheless, it has been proved that the majority of ammonia seen in mouth-exhaled breath has its origin in the oral cavity [47]. Ammonia is also produced systemically, it appears in the body as a breakdown product of proteins, a contribution originated from the bacterial degradation of protein in the intestine [57]. The metabolic pathway is originated in the liver, where the ammonia is converted into urea, which is then eliminated in urine. Some of the ammonia is expelled from the breath and some is emitted by the skin [60].

The metabolic pathways of acetone are well established. The decarboxylation of acetoacetate and the dehydrogenation of isopropanol are the two sources of acetone production [61]. Acetone levels are elevated in diabetes, due to rise of blood sugar level and intensive lipolysis [61]. However, acetone was not reported as a unique biomarker of diabetes. 

Methanol and ethanol may arise as anaerobic fermentation products by gut bacteria [62] including all alcohols in the series from methanol to heptanol. Methanol is contained in some foods, such as apples and drinks, which, when ingested, increases the methanol in the circulation and, hence, in the exhaled breath [62]. Methanol is used industrially as a solvent, pesticide, and alternative fuel source. It also occurs naturally in animals and plants. Methanol can be absorbed into the body by inhalation, ingestion, skin contact, or eye contact. Methanol does not appear to be generated in the mouth and levels detected in breath are of systemic origin. Most breath ethanol, however, appears to be due to mouth fermentation of sugars (unless the subject has been consuming alcoholic drinks) [63].

The biochemical origin of isoprene in human breath is not entirely clear. However, it is considered to be a marker of cholesterol synthesis [64]. Abnormal breath isoprene levels are related to end-stage renal failure and increases in isoprene levels have been associated with oxidative stress. However, this assumption has not been proved by the work of Lirk and co-workers [65]. Little focus has been given, thus far, to the relationships between breath levels and the underlying systemic concentrations. For that reason, King and co-workers [66] joined efforts in investigating the potential stores of isoprene in peripheral tissue groups. Their findings suggested that breath isoprene variability during exercise is linked to local variations of gas exchange in peripheral tissues. The observable wash-out behaviour of isoprene was attributed to an increased fractional perfusion of potential storage and production sites.

2-propanol is a product of the enzyme-mediated reduction of acetone. Bacteria present in the gut produce alcohols, including 1-propanol and 2-propanol, structural isomers that exists in the human body [67].

There is increasing evidence that acetaldehyde, rather than alcohol itself, is responsible for the carcinogenic effect of alcohol [68]. The ethanol levels in the exhaled breath are clearly increased after consumption of sugars and the action on it by either mouth or gut flora/enzymes [59,63]. Acetaldehyde levels result from endogenous ethanol metabolism [36]. As a consequence, acetaldehyde concentrations in breath are invariably lower than the corresponding ethanol concentrations. In healthy individuals, it is rapidly cleared by conversion into acetic acid and thus it is present at low concentrations in the body. However, these levels in breath may not be obvious because acetaldehyde can also be produced from cellular activity involving sugars.

#### 3.2.2. Age Influence/Gender

Age and gender of the volunteer revealed to be an important factor to be taken into account in breath analysis. PTR–MS was used for determination of isoprene concentrations in children’s exhaled breath by Taucher and co-workers, and recognised to be significantly lower than in adults [69]. Lechner and co-workers measured the VOCs on the breath of 126 volunteers, using the same technique, reporting an increase in isoprene concentration of exhaled air of male subjects [70]. SIFT-MS was also used to determine the concentrations of some metabolites in the breath of healthy children aged between 7–18 years old [67]; where the median concentration of pentanol (15 ppb) was also determined. The exhaled breath of several volunteers within the age range 4–83 years was measured and reported a trend of increasing breath ammonia concentration with age [71]. Isoprene is apparently elevated in breath during adolescence, as reported by Smith and co-workers, probably due to the onset of puberty, as stated by the authors [72]. 

#### 3.2.3. Influence of Food

The levels of breath metabolites are influenced by food intake [23,73]. The breath metabolites ammonia, methanol, ethanol, propanol, formaldehyde, acetaldehyde, isoprene, and acetone were quantified by SIFT-MS for a group of five volunteers, before and after ingesting 75 g of glucose in the fasting state [60]. Increased levels in the blood/exhaled breath after the consumption of alcohol were also observed, as reported by Smith and co-workers, using SIFT–MS [63]. Similarly, studies on ethanol metabolism were recently reported by Winkler and co-workers [74]. Metabolic degradation of ethanol was tracked by the ingestion of isotope-labelled ethanol using real-time breath gas analysis with PTR-MS. The findings indicated that in part, ethanol was metabolized to acetone and isoprene, as deuterated acetone and isoprene were observed in the mass spectra. However, the signal of the deuterium-labelled acetaldehyde was not observed, suggesting that this product did not enter the blood stream but was rapidly further metabolized.

The volatile organic compounds emitted by mouth-exhaled breath after garlic ingestion was assessed by PTR-MS, and the main constituents of garlic were reported [75]. The results showed variation in VOCs levels along the time. Other products, such as onion, mint, banana and coffee, are also known to emit volatiles at trace concentrations.

#### 3.2.4. Ovulation

During analysis of the headspace of urine from a number of female volunteers, Smith and co-workers observed acetone and ammonia levels occasionally higher than the normal. The urine samples were collected before any food intake. Such findings suggested that it may be caused by metabolic changes occurring during ovulation and related to menstrual cycle length [76,77].

Studies applied to volatile organic compounds present in exhaled breath correlated with ovulation have not yet been reported.

### 3.3. Exposure to Volatile Compounds

#### Smoking and Air Contaminants

It has been shown that the composition of exhaled breath is considerably influenced by exposure to pollution and indoor-air contaminants, for example, smoking-enhanced acetonitrile concentrations were found in the breath and urine of smokers [3,21,78]. Compounds present in cigarette smoke, such as 2,5-dimethylfuran, acetonitrile, benzene, toluene, and styrene, can also be identified in smokers and passive smokers’ breath [79,80]. Acetonitrile in breath is a good indicator of whether a given subject is a smoker or not because the concentration of acetonitrile in breath takes nearly a week after cessation of smoking to decrease to that of non-smokers [3]. Such findings were reported in an early study using PTR-MS. Smoking increased exhaled ethane and pentane levels in breath. This may be caused by high concentrations of hydrocarbons in cigarette smoke, as well as oxidative damage caused by smoking [81]. Hydrogen cyanide (HCN), along with acetonitrile and benzene, are known to be present in exhaled breath [82], after analysing the exhaled breath of smokers compared with non-smokers. Measuring carbon monoxide (CO) in exhaled breath is a well-established method used to differentiate between smokers and non-smokers [83]. As a constituent of cigarette smoke, carbon monoxide enters the blood circulation during smoking and forms carboxyhemoglobin (COHb). The elimination of CO is primarily by respiration, thus, there is a strong correlation between CO in breath and COHb [83].

Aside from smoking, there are other ways of VOCs entering the body. Breath contains a diverse range of VOCs that can be taken up by the body through inhalation or skin, and, depending on distribution kinetics, may be present in exhaled breath for different periods after exposure. For instance, limonene was found in most air fresheners and cleaning products and is emitted by wooden furniture and floorings. It is known to be soluble in blood and adipose tissue and, therefore, has the potential to be taken up by the body during inhalation [84].

Background contaminants [43,44] are an important issue, particularly, when a compound is present in both alveolar breath and inspired air. One approach is to provide a source of purified air [85] to inspire during the breath collection and, in this way, it is possible to determine which of the VOCs have been endogenously originated. However, different compounds have different wash-out periods from the human breath [86], hence, the use of purified air for removal of exogenous compounds is restricted.

## 4. Sampling and Analysis

The VOCs in breath are at trace levels and that coupled with the high humidity, means that storage and transport of samples is challenging. Therefore, any contamination or improper methodology may have a significant impact on the composition and concentration of VOCs detected in exhaled air. Controlled sampling is a key requirement for reliable analysis of breath biomarkers, because sampling methodology can greatly affect the results. This is a major issue in breath analysis. Now there are no accepted standardized methods for on-line or off-line VOC breath-gas sampling and analysis. The first guidelines concerning sample collection for breath analysis was released in 1999 by the American Thoracic Society for nitric oxide (NO) monitoring in breath [87]. Later on, updated guidelines were published in 2005 for measurement of NO in mouth- or nose-exhaled breath [88]. In 2005, recommendations were also published for exhaled breath condensate sampling and analysis [89]. Hence, reproducibility and reliability of sampling methods and analytical measurement procedures continue to be of critical importance.

Biological variability among subjects has been introduced as an issue in breath sampling. Therefore, breath analysis methods based on monitoring subjects over time may be desirable because they can serve as their own controls [90]. In order to perform breath sampling it is necessary to consider the diffusion of volatile organic compounds from blood to alveolar air, which depends on their physicochemical properties, such as, polarity, solubility in fat, Henry partition constant, and volatility [41].

Temperature dependence strongly influences the composition of breath samples in off-line measurements. As the temperature of breath sample falls below body temperature (37 °C), water vapour condenses on the inside of the bag and takes down water soluble compounds. Warming the sample to body temperature will avoid condensation issues and compound losses due to negative temperature gradients [91]. Further aspects related to sampling continue to be debated in the scientific community, such as body posture of the subject when providing the breath sample; hyperventilation; control of the flow or volume of breath during collection; sampling via nose or mouth; number of breath samples to be taken to reduce variability (single or multiple breaths); dilution and contamination of the sample; physiological parameters, such as respiratory rate or heart beat rate; alveolar breath or end-tidal volume and dead space; number of subjects per study to avoid over-modelling; and direct analysis or sampling for storage [90].

The number of issues to be considered suggests that the development of several protocols for standardized breath sampling may become mandatory.

### 4.1. End-Tidal and Alveolar Breath

Concentrations of volatile compounds in blood are reflected by their concentrations in the exhaled air, depending on their blood-gas partition coefficient or solubility. Alveolar breath is the part of exhaled air in equilibrium with systemic blood, whereas end-tidal air is the last fraction of expired air, whose composition resembles alveolar air. Generally, the term end-exhaled breath is applied because it does not imply that the composition of expired air is always identical to the equilibrated air inside the alveoli. It has long been acknowledged that alveolar gas exchange is dependent on ventilation, pulmonary perfusion, and the blood:air partition coefficient [92], thus, the non-homogeneities in the composition of alveolar air among different lung regions over different blood:air solubilities of volatile organic compounds [93]. There is evidence that the gas exchange of highly soluble volatile compounds occurs in the airways rather than alveoli [94], meaning that VOCs measured at the mouth depend on expiratory flow. Generally, the term alveolar breath may be applied for low blood soluble VOCs, whereas for highly soluble volatiles such as acetone the term end-exhaled breath should be used due to the evidence that gas exchange occurs in the airways rather than alveoli. Such evidence was quantified for the first time by Španěl and co-workers, who demonstrated the discrepancy between the concentration in the alveolar region to that in exhaled air (a factor of 3 for isoprene) [44].

Breath analysis for medical diagnosis relies on end-tidal sampling [95], involving the collection of only end-tidal air. Alveolar concentration reflects the concentration in blood and consequently, the concentration in blood reflects the metabolic processes occurring in the body. Furthermore, there is evidence that better reproducibility of data is obtained when only the end-tidal fraction of breath is analysed [96].

Earlier in 1948, it was noted by Fowler that the volume of exhaled air is a mixture of dead space and alveolar air [97]. Dead space was previously defined as the volume of expired air, which acts as a conducting airway (nose, pharynx, larynx, trachea), whereas alveolar air is the expired air fraction that has been exchanged in the alveoli.

Initial approaches have been taken, such as discarding the first 500 mL of exhaled breath to avoid dilution of the sample by dead space. However, it incorrectly assumes that all subjects have the same volume of dead space [98].

Monitoring of expired CO_2_ has been used to identify alveolar gas. Thus, Schubert and co-workers measured CO_2_ [99,100] in exhaled air of mechanically ventilated patients by means of a capnograph, using a CO_2_‑triggered alveolar sampling valve. They reported this as a reliable and reproducible method for alveolar sampling through CO_2_ concentration measures. Later on, Di Francesco and co-workers designed a CO_2_-triggered breath sampler suitable for multiple breaths [96]. More recently, Filipiak and co-workers [101] applied the same on-line monitoring of expired CO_2_ in order to collect alveolar air by needle traps used in GC-MS analysis.

The on-line breath sampling so-called *buffered end-tidal* (BET) [95] breath sampling method has been developed to extend the analysis time of the end-tidal fraction of a single exhalation. This sampling system was designed to buffer only the end-tidal fraction of the breath. The patient is asked to exhale through a tailored tube in which the end-tidal fraction of breath is buffered. 

Concentration of breath molecules, prior to mouth appearance, still remains a hard task to unravel due to the lack of knowledge about the parameters and processes which could affect the VOCs’ final concentrations in the mouth. For instance, highly soluble compounds are diluted on their way up from the deeper respiratory track to the airway opening, leading to a dilution effect on VOCs’ concentrations. Such an important subject is further explained in Section 4.2. The concentrations of breath molecules exhibit flow rate dependency, namely, changes in ventilation strongly influence quantification of volatiles in exhaled breath [102]. In additional, body posture and stress can have a significant impact on the observed breath concentration [102]. Breath holding [93] has been demonstrated to significantly increase the exhaled concentrations of breath gases (H_2_, CH_4_, and CO).

These sampling systems, which selectively extract end-tidal air by discarding anatomical dead space volume, are far from being perfect, since they do not take into account physiological variability.

### 4.2. Dilution and Contamination

Hydrophilic exhaled trace gases, such as acetone, interact with the water-like mucus membrane lining the conductive airways, an effect known as wash-in/wash-out behaviour [103]. The exhaled breath concentrations of water soluble substances appear to dilute on their way up from the deeper respiratory track to the airway opening, leading to discrepancies between the true alveolar breath and the measured concentrations, demonstrating a dilution effect. It means that highly soluble gases are present in large concentrations in the airway tissue and mucus as compared to less blood-soluble gases for a given partial pressure. An absorption-desorption phenomenon occurs in the airways; this is firstly initiated by absorption of soluble gases from the airway wall to inspired air, during inspiration. By the time the air reaches the alveoli, the air is saturated with soluble gas and no further gas exchange occurs. During expiration, a gradient air-to-mucus is established promoting the deposition of soluble gas on the mucus and delays the rise in soluble gas partial pressure at the mouth. An anatomic dead space cannot be defined for these gases [92,94]. 

The airway gas exchange is influenced by perfusion; diffusion through the airway wall; and temperature. Perfusion is driven by the bronchial blood flood, meaning that an increase in blood flow increases the amount of blood soluble gas in the exhaled breath. 

Smith and co-workers recently reported a quantitative study, where they investigated the relationship between the exhaled and inhaled air concentrations for seven compounds [44]. The volunteers were deliberately exposed to known concentrations of some compounds, within the range of permissible exposure limits. Their findings were consistent with previous models, and the equilibrium concentration of acetone was shown to be enhanced above the measured exhaled end-tidal value by 19% for all subjects.

### 4.3. Sampling of Single or Multiple Breaths

Breath sampling may be performed for a single breath or for multiple breath cycles [95,101,104]. However, the composition of a single breath may not be a representative alveolar gas sample for the reason that breaths may considerably vary from each other due to different modes and depth of breathing. Multiple breaths may be preferable in order to acquire reproducible breath samples. However, it may be that exact quantification is less important when characterizing breath profiles. Comparison of rebreathing and on-line single exhalations of highly soluble compounds acetone and methanol, and the low soluble isoprene, was previously evaluated [104,105]. For highly soluble compounds, such as acetone, exchange occurs in the airways rather than alveoli [94], thus, breath sampling becomes much more complicated because acetone concentration in end-exhaled breath may not be in equilibrium with the systemic blood. For that reason, isothermal rebreathing model has been proposed for estimating the alveolar levels of highly soluble exhaled endogenous volatiles [105].

Breathing patterns [102] have been studied and measurements, such as mouth pressure, tidal volume, respiration rate, end-tidal carbon dioxide, and mixed expired carbon dioxide, were recorded. Paced breathing [102] profiles showed reduced breath variability, according to mass and respiration rate. The authors suggested that controlled breathing would prevent hyperventilation, reducing variability in ventilation. 

### 4.4. Storage and Stability of Breath Samples

Direct sampling is preferable to storage for later analysis. This way the decomposition of samples or loss of compounds by diffusion is avoided.

When direct analysis is not possible, the appropriate storage of exhaled breath is an important issue to consider. Background emission of pollutants, losses by diffusion through the bag or adsorption to the inner bag, and interactions between sample constituents, namely reactive chemistry of the stored sample, may irreversibly modify the original sample composition and consequently distort the final results of analyses. 

Currently, Tedlar bags are the most common materials for breath collection [106]. Nalophan bags are also popular due to its low price, inertness, and relatively good durability. Generally, breath may be stored in several ways [51]:

- Transparent or black Tedlar bags (PTFE-polytetrafluoroethylene)
- Flexfoil bags (PET/NY/AL/CPE-polyethylene terephthalate/nylon/aluminium foil/chlorinated polyethylene)
- Nalophan bags (PET-polyethylene terephthalate)
- Glass vials (for SPME)
- Thermal desorption tubes (different adsorbents, used in TD GC-MS)
- Micropacked sorbent traps
- Metal canisters

The stability of selected breath constituents in polymer sampling bags have been previously investigated and assessed by PTR-MS and GC-MS [106,107,108]. Smaller samples are more vulnerable to VOCs losses by permeation. Additionally, the volume of the sample collected affects the stability of the sample, thus, Mochalski and co-workers recommended sample collections as large as possible to prevent background emissions of contaminants [106]. Previous studies reported the testing of Nalophan bags for storing tobacco samples, and investigated the factors contributing to decay of gas samples during storage, between 4 and 40 h after collection [109]. Samples remained relatively stable between 4 and 12 h after sampling. The odour concentration decreases after 30 h storage to about half of their initial value, due to diffusion effects. Background contaminants released from the bags must be taken into account. Emissions from Nalophan, Flexfoil and Teflon bags were assessed 48 h after filling for storage of volatile sulphur compounds, and none showed emissions of contaminants, thus, all proving to be excellent materials for breath sample storage. Tedlar bags, however, showed significant emissions of COS and CS_2_, especially for black Tedlar bags with COS and CS_2_ emissions being seen at up to 7 ppb after three days of storage [108]. A study performed by Gilchrist and co-workers investigated the collection and storage of breath samples containing hydrogen cyanide [91]. Breath was collected into 25 µm thick Nalophan, 70 µm Nalophan and Tedlar bags, at 20 °C or 37 °C. Results showed better correlation between on-line and off-line concentrations for all bag types at 37 °C. Correlation of hydrogen cyanide concentrations in breath samples stored at 37 °C was good up to 24 h for the 70 µm Nalophan and Tedlar bags. Such findings suggested that either would be appropriate to use for collection of breath containing hydrogen cyanide. However, Nalophan bags are much cheaper than the Tedlar bags and they can be discarded after a single use, removing the need for bag cleaning or infection control measures. 

Humidity also affects the species recoveries, and the high humidity in exhaled breath might cause significant decrease in vapour concentrations for those compounds highly miscible with water [106,107]. Water vapour diffuses through most bags at a speed dependent on the temperature of the bag material [91]. Such findings can easily be tracked by exploiting the full capabilities of SIFT-MS to measure the water vapour in air/breath samples. 

Condensation affects the sample authenticity, especially for water-soluble compounds. The loss of volatile compounds to condensed water in Tedlar bags used for breath sampling has been previously evaluated [110] showing differences between dry and wet matrices smaller than 10%. For VOCs with molecular mass above 110 amu, higher losses were detectable (20%–40%) [106]. Thus, Mochalski and co-workers recommended storing breath samples in pre-conditioned Tedlar bags up to 6 h at the maximum possible filling volume. 

Recently, needle trap micro-extraction (NTME) [111] combined with GC has been assessed for sample preparation in VOCs analysis. This is a technique similar to SPME but with the advantage of allowing automatic alveolar sampling. The analysis is quite similar to that used in thermal desorption traps. VOCs are thermally desorbed from the needle trap device and separated, identified and quantified by means of two-dimensional gas chromatography combined with MS detector, GC × GC-MS, which has been applied to solve complex problems of separation. Needle traps have offered increased robustness in comparison to SPME, due to the existence of an extraction sorbent packed inside a hypodermic needle rather than supported on a fragile silica fibre that is exposed to the breath matrix during extraction. The influence of humidity, sample volume, and sampling flow has to be thoroughly evaluated in order to be used for pre-concentration of breath volatiles.

### 4.5. Physiological Parameters

The complex physiological mechanisms underlying pulmonary gas exchange makes breath analysis a challenging subject. Gas exchange [112] during respiration occurs primarily through diffusion. It takes place between the air within the alveoli and the pulmonary capillaries. 

Nowadays, it is known that exhalation of breath biomarkers may well depend on physiological parameters, such as blood pressure, heartbeat rate and alveolar ventilation [113]. Exhaled acetone concentrations mirrored exercise induced changes of dextrose metabolism and lipolysis [113]. Understanding the influence of these factors is an essential requisite for the development of a reliable methodology based on breath volatiles. Breath gas concentration can then be related to blood concentrations via mathematical modelling. The simplest model relating breath gas concentration to blood concentrations was developed by Farhi. 

Through Farhi [114], Equation (1), is observed that alveolar air concentration, C_A_, is proportional to the concentration of VOCs in mixed venous blood (C_V_) and depends on: blood:air partition coefficient, λ_b:air_, which describes the diffusion equilibrium between capillaries and alveoli, and ventilation-perfusion ratio, $\frac{V_{A}}{Q_{c}}$. Such ratio ensures that the ideal amount of blood and gas is received by the alveoli for efficient gas exchange. It depends on the alveolar ventilation (V_A_) controlling the transport of the VOC through the respiratory track, and cardiac output (Q_c_) controlling the rate at which the VOC is delivered to the lungs. Blood:air partition coefficient, λ_b:air_, is strongly dependent on temperature ranging from 23 °C in the mouth to 37 °C in the alveoli, affecting soluble gas exchange [92]. This coefficient represents the ratio of the concentration in blood to the concentration in the gas phase.

(1) $$C_{measured\;}=\;C_{A}\;=\frac{C_{V}}{\lambda b:air\;+\;\frac{V_{A}}{Q_{c}}}$$

For low blood soluble gases (λ_b:air_ ≤ 10) [92] the measured concentration is dependent on the rates at which blood is pumped through the lungs and ventilation, specifically the ventilation-perfusion ratio, $\frac{V_{A}}{Q_{c}}$, where C_measured_ = C_A_, meaning that low blood soluble VOCs must exchange completely in the alveoli.

Highly soluble VOCs (λ_b:air_ > 10) [92] tend to be less affected by changes in ventilation and perfusion, however, hydrophilic exhaled trace gases, such as acetone, interact with the water-like mucus membrane lining the conductive airways. The exhaled breath concentrations of these volatiles appear to dilute on their way up from the deeper respiratory track to the airway opening (dilution effect), consequently for these highly soluble volatiles the concentration measured in exhaled breath is different from the alveolar air concentration, C_measured_ ≠ C_A_. There is also evidence that, with highly soluble volatile compounds, gas exchange occurs in the airways rather than alveoli [94].

Some studies have been performed assessing the concentration profiles during exercise [66,113,115,116,117]. Recently, the influence of exercise on mouth-exhaled and nose-exhaled breath was further investigated [117]. Smith and co-workers reported significant increase of isoprene breath levels which are in agreement with previous findings [66].

An isoprene gas-exchange model (2) was developed and showed good fit to breath isoprene levels measured during exercise. Dependency of heartbeat rate and breath rate for isoprene breath concentrations have been assessed, where isoprene levels were measured during exercise [113,116]. Isoprene concentrations showed drastic increase within the initials seconds of exercise [113,116], followed by a decline when heartbeat rate reached the maximum value and respiration rate increased, and lastly at the end of the exercise isoprene concentrations reached similar levels seen at the beginning. This means that the degree of blood-to-air partitioning of isoprene is very sensitive to heart rate. Such measurements demonstrates a relationship between breath rate volume (V*br*), heartbeat volume (HBV), Henry’s law constant (*H*) and temperature (T), seen in Equation (2) [116].

(2) $$C_{A0\;}=\;C_{V0}\;\cdot \;exp\left(-\frac{1}{HRT}\;\cdot \;\frac{Vbr}{HBV}\right)$$

For volatiles such as isoprene, with low solubility in blood and high volatility (Henry’s law constant extremely low) a concentration gradient within the lungs is created and governed by the velocity of the bloodstream pumped through the lungs (proportional to heartbeat frequency) and the breathing rate. Namely, with increases in both heart rate and breathing rate, more efficient partitioning of isoprene to breath air is restored. This means that isoprene evaporates efficiently through the transport via the bloodstream to the lungs, hence, C_A0_ ≠ C_V0_, meaning that isoprene venous blood concentration entering the lungs, C_V0_, is different from isoprene arterial blood concentration leaving the lungs, C_A0_. 

Moreover, measures taken during sleep showed enhanced blood isoprene concentration due to lower heartbeat rate achieved during the night [116].

## 5. Disease Diagnosis

### 5.1. Volatile Biomarkers

Current cancer detection methods include Computer tomography (CT) scanning, magnetic resonance imaging (MRI), as well as biopsies. However, several cancers such as lung cancer, colorectal cancer, bladder and prostate cancer are very difficult to detect at an early stage due to the lack of sensitivity of those methods. SIFT-MS and PTR-MS have, therefore, been used to determine whether they have potential for identification of possible cancer biomarkers. Other applications include diagnosis in liver disease, infectious diseases, food intolerances, and monitoring of diabetes.

#### 5.1.1. Lung Cancer

Lung cancer is the most common cancer and it has one of the lowest survival outcomes of any cancer because over two-thirds of patients are diagnosed at a late stage when curative treatment is not possible. Methylated hydrocarbons are proposed for lung or breast cancer biomarkers [1].

Nowadays, it is well known that acetaldehyde is present in breath of healthy people [59] at a physiological mean level of about 22 ppb. Acetaldehyde above physiological levels in exhaled breath could have major clinical importance. However, these levels in breath may not be obvious in most of the cases. Acetaldehyde is an intermediate in the metabolism of ethanol in the liver, however, intake of alcohol will greatly elevate acetaldehyde levels in breath [63]. In addition, acetaldehyde can also be produced from cellular activity involving sugars.

Phillips and co-workers suggested, in a cross-sectional study, a combination of 22 VOCs potential markers of lung cancer, in breath samples of patients with and without lung cancer [4].

To support lung cancer studies, cells *in vitro* studies were performed in order to analyse the molecular emissions from cancer cells lines SK-MES and CALU-1 [118]. The experimental results showed that acetaldehyde is present in the headspace above cell cultures at levels significantly higher than physiological levels, a potential lung cancer biomarker. A contradicting publication reported by Filipiak and co-workers, using cell lines CALU-1 which volatiles were analysed by GC-MS, showed that compounds are not released but seem to be consumed by CALU-1 cells. These findings confirmed the existence of compounds that are either released or consumed by these cells [119]. Nevertheless, do the cell lines growing *in vitro* have similar characteristics to *in vivo* cancer cells? A 3D model has been proposed [120] in which the cells were cultured in 3D scaffolds composed of collagen type I hydrogels, compared to 2D models where cells are grown on surfaces such as plastic or glass. Quantification by SIFT-MS of cells lines headspace CALU-1 and non-malignant lung cells NL20 revealed that the amount of acetaldehyde released by both cell types grown in a 3D model is higher when compared to that of the same cells grown in 2D models.

#### 5.1.2. Colorectal Cancer

Colorectal cancer has been attributed to individual genetic predisposition and environmental factors, including lifestyle and diet. Within lifestyle factors, elevated body mass index (BMI), obesity, and low physical activity are related to increased risk of colorectal cancer. It has been shown that diet can significantly influence and promote the growth of malignant colon cells, particularly, red meat intake, where protein is the major constituent leading to protein fermentation metabolites potentially carcinogenic and possible linked to colon cancer [121,122,123,124]. Therefore, diet may have complex effects on the generation of breath compounds [125]. The findings indicated that diets low in fat and rich in protein induced systemic ketosis leading to increased levels of acetone in breath [126]. Acetone may be reduced to isopropanol by hepatic alcohol dehydrogenase and, consequently, it may appear in the breath [125]. Carbohydrate fermentation by bacteria in the gut results in production of hydrogen, methane [127], carbon dioxide, and short chain fatty acids (SCFAs) mainly acetate, propionate and butyrate as the main non-gaseous fermentation end products. Early measurements of hydrogen in breath have been used to study carbohydrate absorption in the small intestine [128]. Similarly, measurements of methane in breath have been used to assess colonic bacterial metabolism [129]. Short chain fatty acids are assimilated by the host and used for the energy metabolism. Butyrate in particular [130] has been considered to have a protective role, protecting against colitis [130] and colorectal cancer [131]. Hence, bacteria have an important role on the generation of the majority of some compounds present in breath, such as hydrogen, hydrogen cyanide, aldehydes, and alkanes [125]. Recently, Schmidt and co-workers reported that breath hydrogen cyanide may rise following the consumption of food or drink [132]. Ammonia, isovaleric and isobutyric acid (BCFA), phenolics and hydrogen sulphide have been identified in breath as products of gastrointestinal bacterial fermentation. 

Pentane, ethane and ethylene have also been identified in breath as products of increased lipid peroxidation [125]. 

Recently, Altomare and co-workers used GC–MS to analyse breath samples from patients with colorectal cancer, and concluded that the pattern of VOCs in patients suffering from colorectal cancer were different from that in healthy controls, particularly levels of some specific VOCs such as 1,3-dimethylbenzene, 1,2-pentadiene, cyclohexene and methylcyclohexene [133]. However, further studies are necessary to support this experiment. 

Normal metabolism generates VOCs that may emanate from faecal matter. Human faecal flora comprises bacteria involved in colonic fermentation producing sulphur containing compounds, such as hydrogen sulphide, dimethyl disulphide, methyl disulphide, and dimethyl trisulphide, and these compounds are responsible for the specific odour of faecal matter [134]. Thus, analyses of human metabolites as end products of intestine may rely on faecal samples or on breath. 

#### 5.1.3. Breast Cancer

Breast cancer is accompanied by increased oxidative stress caused by lipid peroxidation of polyunsaturated fatty acids in membranes, producing alkanes and methylalkanes, such as 3-methylundecane, 6-methylpentadecane, and 2-methylpropane, among others, potential biomarkers as suggested by Phillips and co-workers. Hietanen and co-workers performed an initial case-control study where they analysed the breath of women with breast cancer and found increased concentrations of pentane [135]. Nevertheless, the majority of the investigations of volatile biomarkers potentially used in breast cancer diagnosis has been done by Phillips and co-workers. They performed a pilot study of breath VOCs in women with breast cancer. Breath samples were analysed by GC-MS and compared with abnormal mammograms and biopsies. The breath test distinguished between women with breast cancer and healthy volunteers with a sensitivity of 94.1% [136]. Their recent findings were consistent with previous studies, however the biochemical origin of volatile biomarkers of breast cancer remains speculative [8]. 

#### 5.1.4. Liver Disease

Few studies have been performed in order to use breath analysis as a screening tool for liver disease diagnosis. Sulfur-containing compounds, such as dimethylsulfide, hydrogen sulphide, and mercaptans (e.g., methylmercaptan and ethylmercaptan) are proposed as liver cancer biomarkers [1].

Ammonia levels rise in the blood when the liver is unable to convert ammonia to urea. This may occur because of cirrhosis or severe hepatitis, however the majority of ammonia seen in mouth-exhaled breath is largely generated in the oral cavity [47]. Poor oral hygiene can be a confounding factor, because production of ammonia from urea may increase the ammonia levels in exhaled breath. Such a situation may be mitigated by mouth washing thoroughly with water before breath sampling [50]. Very little information is available about the possible use of VOCs in patients with liver cirrhosis. An initial study performed by Van den Velde and co-workers [137] showed discrimination between the cirrhotic group and healthy subjects. They analysed the breath of 50 patients with established liver cirrhosis. Recently, a pilot study using PTR-MS equipped with a time-of-flight mass analyzer was conducted in liver cirrhosis patients by sampling the breath of the subjects [138]. The authors were able to distinguish between healthy and disease subjects. They identified twelve different VOCs significantly different between cirrhotic and healthy subjects.

Hepatic encephalopathy [139] is a neuropsychiatric syndrome with symptoms varying depending on the severity of the condition. It results from the accumulation of compounds not cleared by the liver. Ammonia is known to be involved in hepatic encephalopathy [139], however attempts to use breath ammonia measurements for diagnosis have failed, probably because most exhaled ammonia is generated within the oral cavity by bacterial and/or enzymatic [47]. Additionally, some confounding factors have proven to be tricky in VOCs detection and quantification. For example, pulmonary gas exchange abnormalities can be present in patients with advanced liver disease, such as high cardiac output and abnormal dilation of pulmonary capillary vessels, leading to incorrect conclusions [139].

#### 5.1.5. Infectious Diseases–Tuberculosis

Pulmonary tuberculosis (TB) is an infectious disease derived from the organism Mycobacterium tuberculosis. The primary detection technique is the Ziehl-Neelsen staining combined with microscopy. It only allows detection of pulmonary disease in an advanced stage, meaning that often the disease has already been transmitted to close contacts.

Breath analysis may offer a method for diagnosing pulmonary tuberculosis [140]. Some compounds potential biomarkers have been found by breath sampling, such as methyl phenylacetate, methyl *p-*anisate, methyl nicotinate, and *o*-phenylanisole [141]. However, contradictory studies presented different marker compounds, possible due to the fact that *Mycobacterium tuberculosis* is a slow growing organism. This means that if VOCs are produced they may be released or modified by the host, hence, they may well be present at low concentration and not be detected by the real-time analytical techniques. The best approach should be looking at clinical samples in order to get potential biomarkers of pulmonary tuberculosis [36].

#### 5.1.6. Food Intolerances

Other breath analysis studies have been performed such as from people suffering from carbohydrate malabsorption, a condition in which the patients are unable to absorb or digest certain carbohydrates due to the lack of some intestinal enzymes, leading to bacterial sugar fermentation in the gut. Coeliac disease is an under-diagnosed autoimmune disease of the small intestine characterized by nutritional malabsorption, for which was conducted a preliminary investigation of the levels of alcohols in the breath of 10 patients with coeliac disease compared to that in 10 healthy controls using SIFT-MS [142]. No significant conclusions were drawn. Such conclusions are in agreement with recent findings performed by Aprea and co-workers [143], where real time breath analysis was performed in patients diagnosed with coeliac disease under gluten free diet. As expected, exhaled breath of patients with coeliac disease was similar to the exhaled breath of healthy people and no reliable marker was found. 

A novel approach to the diagnosis of gastro-intestinal diseases was attempted by Lechner and co-workers using PTR-MS, through headspace screening of fluid obtained from the gut during colonoscopy and analysis of exhaled breath, either from healthy controls and patients suffering from inflammatory bowel disease (IBD) and irritable bowel syndrome (IBS) [144]. Fluid samples of patients with IBD showed enhanced peaks at *m/z* = 57 and *m/z* = 83, while no significant differences were found for IBS patients group. Further comparison of breath samples revealed increased concentration of ions at *m/z* = 31 and *m/z* = 77 in the IBS group. Lechner and co-workers suggested that the ions detected at *m/z* = 31 and *m/z* = 77 probably represent protonated formaldehyde and protonated acetone with an attached water molecule respectively [144]. However, the use of *m/z* values for biomarker detection is unreliable, especially at higher *m/z*, as ions could represent any or many of a number of compounds. Recently, Dryahina and co-workers [145] reported pentane as potential biomarker of bowel disease, by analysing the breath of patients with Crohn’s disease and ulcerative colitis and of healthy volunteers, in a pilot study using SIFT-MS.

## 6. Clinical Studies

### 6.1. Diabetes Mellitus

Centuries ago, John Gallo reported a compound in human breath that had the smell of decaying apples [146]. Nowadays, it is well known that the compound was principally acetone [147]. Acetone is produced by decarboxylation of acetoacetate and through dehydrogenation of isopropanol [61]. Diabetic patients exhibit increased concentrations in blood and urine of ketone bodies, acetone, acetoacetic acid, and beta-hydroxybutyric acid. Ketone bodies are produced by the liver during fatty acid metabolism, and are used as an energy source, instead of glucose, when glucose is not readily available [148].

Acetone is a highly soluble gas present as a major common breath metabolite in everyone. This volatile organic compound has been identified and quantified previously [40,57]. Elevated breath acetone levels were early associated with diabetes mellitus [61,149,150].

A few studies have been carried out, involving cohorts of patients with diabetes and healthy controls, in who parallel blood glucose levels have been determined. Biochemical changes that occur in the disease state (diabetic state) may be reflected in changes in the profile of VOCs in exhaled breath. The high inter-individual variability in breath acetone concentration is not well understood. Contributors may include diurnal variability, fasting status, diet, age, and gender.

Acetone levels were previously quantified for healthy volunteers, before and after ingesting 75 g of glucose in the fasting state [60]. The intake diet seems to significantly influence the levels in breath acetone. Measurements were taken following a ketogenic diet [126,151] in the exhaled breath of healthy individuals, and for a small group of individuals suffering from diabetes. Results have shown that breath acetone concentrations increased after ingestion of a ketogenic meal, or either following a low carbohydrate diet [151]. Smith and co-workers reported that breath acetone increases substantially during fasting when a change takes place from carbohydrate to fat metabolism [148]. Schwarz and co-workers, using PTR–MS, reported a variation on breath acetone with age and fasting state, but no statistically significant differences between gender or body-mass index (BMI) were found [152].

Glycaemic control is essential for management of diabetes. At the moment, blood analysis is the faster way to provide information/monitoring of glucose levels in patients with diabetes. However, studies amongst adults showed discomfort associated to blood sampling and needle phobia [153,154]. Only recently, breath analysis appears as a potential non-invasive method for monitoring glucose concentrations in blood [148,155,156]. Turner and co-workers have monitored the breath of eight patients with type 1 diabetes mellitus using a glucose clamp technique [156]. In all patients, the breath acetone declined linearly with blood glucose concentration. Hence, this study indicates that breath acetone does vary as glycaemia and/or metabolic status changes in type 1 diabetes.

Compounds present in exhaled breath displayed very strong correlations with glucose concentrations, in another study conducted in healthy and type 1 diabetic subjects [157]. Standard least squares regression was used on several subsets of exhaled gases to generate models to predict plasma glucose for each subject. 

Little is currently known for type 2 diabetes, some measurements of breath acetone in type 2 diabetes were taken, nevertheless, with no significant results [158].

A recent study performed by Righettoni and co-workers reported the correlations between blood glucose of healthy volunteers and breath components, from portable gas sensors and PTR-MS equipped with a time-of-flight mass analyzer [159]. The relationship between the PTR-MS measurements of breath gases acetone, isoprene, ethanol and methanol, sensor response and the blood glucose level was studied. They reported a better correlation between blood glucose level and breath acetone for the overnight fasting (morning). 

Breath composition during oral glucose tolerance tests was analysed by TD GC-MS in 16 subjects and correlated to blood glucose levels [160]. The glucose tolerance tests classified five of the subjects as diabetics, eight as affected by impaired glucose tolerance, and three as normoglycaemic. A clustering algorithm was used to differentiate individuals between groups based on blood glucose values at different times, and breath acetone concentrations. Acetone levels were generally higher in diabetics. 

Dogs have been used to detect hypoglycaemic episodes in their diabetic owners through detecting breath or skin odour [161].

Isoprene has also been proposed as a potential indicator of diabetes [162]. However, several studies reported no apparent correlation between blood glucose and breath isoprene [58,163]. Methyl nitrate is also suggested to be correlated with blood glucose in insulin dependent diabetics, though the levels are reported to be lower than acetone or isoprene. Therefore, this might not be a useful compound for monitoring purposes [148].

Although the findings have pointed to acetone as potential biomarker of diabetes, there is no simple association of breath acetone concentration and diabetes. The issue lies in the fact that acetone generation is linked to lipolysis and blood glucose changes. 

## 7. Biomarkers *vs*. Biomarker Profiles

The complex relationships between a number of different compounds and the presence or absence of a disease or condition indicates that perhaps volatile biomarkers profiling with bioinformatics is a more promising approach. A specific breath marker related to a specific disease is the ideal. However, this is unlikely to be the case for the majority of diseases or conditions, where it is more probable that a range of VOCs with varying concentrations will have to be used. By adopting a strategy of identifying patterns, rather than trying to identify individual VOCs, “breath fingerprinting” could provide a suitable and reliable method for discriminating between healthy and diseased states. This approach requires elaborate methods of data analysis, pattern recognition techniques, such as principal component analysis (PCA) and partial least squares discriminant analysis (PLSDA). Principal component analysis is a mathematical algorithm that reduces the dimensionality of the data. It accomplishes this reduction by identifying directions, called principal components, in which the variation in the data is maximal. Samples can be plotted, and visually assess similarities and differences between samples, and determined whether samples can be grouped. Other multivariate methods also exist, such as PLSDA, or support vector machines (SVMs). All of these methods use whole profiles, and yet it is possible to identify individual components (e.g., compounds or ions), which are most responsible for the differences observed between groups (e.g., groups of samples positive or negative for a disease). Thus, “biomarkers” (which are not usually unique) may be identified in this way. Cross validation of the models is used to predict the classification capabilities on unknown objects. Hence, if there is a good correlation between the predicted and actual values this means that the model fits. 

In clinical practice, biomarkers such as genes and proteins are identified and quantified in order to track the biochemistry within the body for a specific disease. However, clear quantification of VOCs is a harder job due to the difficulties in finding the biochemical pathways in the body for each metabolite. This will involve a close collaboration between clinicians and analytical chemists. 

The need to understand the relationships between many variables makes multivariate analysis an inherently difficult subject. It is important to note that when the number of variables quickly overwhelms the number of samples, spurious correlations may be found [164]. Confounding variables have a real statistical correlation with the disease and a breath marker, leading to wrong conclusions. Confounding variables comprise environmental compounds, physiological parameters and even the sampling procedures [164]. The statistical technique used to control the influence of confounding variables is called Analysis of covariance (ANCOVA). Classification of the subjects into groups is achieved by discriminant analysis, cluster analysis, and propensity score analysis. Clustering attempts to find similarities among the subjects that were measured instead of among the measures that were made. For multiple dependent variables, in which two or more dependent variables are included, multivariate analysis of variance (MANOVA) and canonical correlation analysis are applied. Recently, Halbritter and co-workers [165] used MANOVA technique to discriminate according to whether the pregnant women had gestational diabetes mellitus, impaired glucose tolerance, or normal glucose tolerance, by means of analysing the women´s breath by PTR-MS and correlating it with the oral glucose tolerance test. 

The success of proper statistical analysis is to have a good statistical validation, as well as trustworthy biological interpretation of the results. 

## 8. Breath Test as a Clinical Diagnostic

Initial pilot exploratory studies aimed to establish the population distributions of the metabolites levels in breath of the healthy population [55]; to study the enhancement of breath metabolites by drug ingestion [166]; to investigate the influence of smoking on the breath metabolites [3,80]; among others. For such studies, the sample size of volunteers is not a restriction. 

The concepts of standard error and confidence interval should be highly understood in order to determine the sample size of patients needed to develop a diagnostic test. The need of power calculations to calculate the minimum sample size required should be used. The sample size determines the amount of sampling error inherent in a test result. Effects are harder to detect in smaller samples leading to increased standard errors. Increasing sample size is often the easiest way to boost the statistical power of a test. A common recommendation by statisticians calls for ten times as many subjects as the number of independent variables. Great concern should be taken about sample size of controls and subjects with the disease. However, several studies performed up to date used small control groups or/and small groups with the disease, due to the difficulty and cost of obtaining a significant sample size of subjects. Nevertheless, such strict rules are not required for essential pilot exploratory studies, as may be needed eventually. Moreover, the selection of appropriate controls is an important issue to take into account. An ideal control group should not have the disease in question, but be comparable to the diseased group, for instance have identical symptoms. 

Until now, the clinical issues are far behind the analytical techniques, which have been proven to be extremely accurate and sensitive for trace gas analysis. As proven by the extensive studies of hydrogen cyanide (HCN) in relation to *Pseudomonas aeruginosa* infection, performed by Smith and co-workers by SIFT-MS [167]. They reported hydrogen cyanide as a volatile biomarker of *Pseudomonas aeruginosa* infection. 

There is the need for identification of the relationships between biochemical pathways and disease; normal ranges and limiting concentrations for the breath VOCs for healthy subjects; and variations of VOCs concentrations with age, gender, and ethnicity. Along with identification of the minimum concentrations of biomarkers indicative of disease, the concentration of biomarkers, which identify the stage of disease, should be known as should the variations of abnormal concentrations with age, gender and ethnicity. Furthermore, clinical diagnostic tests using breath must be suitable for all categories of patients, namely children, adults, and elderly patients, and sensitivity and specificity be determined. Particular attention must be paid to patients with disabilities, ventilated patients, and asthmatic patients. The breath test must be as accurate as the existing screening tests, cheaper, quicker, and non-invasive to patients. 

The clinical significance of the breath test will determine the applicability of it into clinical practice.

## 9. Concluding Remarks

Detection of volatile compounds in breath at trace concentrations can be an indicator of metabolic status, allowing identification of diseases in their early stages. Breath analysis has proved to be suited for the diagnosis of cancer, infectious diseases, food intolerances, and diabetes, among others. However, the complexity of VOCs in exhaled breath makes breath a difficult sample to analyze and the findings are not always consistent. Therefore, it has been difficult to reach an agreement regarding the identification of volatile biomarkers. 

In order to measure the concentration of a volatile compound in exhaled breath, some understanding of the compound’s exhalation physiology is necessary. Important factors, such as mouth generated volatiles, pulmonary gas exchange, contamination, *etc*., should not be neglected. To introduce breath analysis into clinical practice, sampling procedures have to be standardized and the metabolic pathways clearly understood. In addition, factors that are unrelated to disease, but capable of changing the concentration of a volatile compound in exhaled breath must be well understood in order to develop robust clinical tests. The findings lead to the suggestion that the creation of a unique diagnostic test would not be the best option. Instead, the creation of a diagnostic test according to the disease would be the best approach. 
